# Inflammatory Bowel Disease from the Perspective of Newer Innate Immune System Biomarkers

**DOI:** 10.3390/gidisord7010022

**Published:** 2025-03-06

**Authors:** Martin Tobi, Fadi Antaki, MaryAnn Rambus, Jason Hellman, James Hatfield, Suzanne Fligiel, Benita McVicker

**Affiliations:** 1 Department of Research and Development, Detroit John D. Dingle VAMC, Detroit, MI 48201, USA; 2 Research Service, VA Nebraska-Western Iowa Health Care System, University of Nebraska Medical Center, Omaha, NE 68105, USA

**Keywords:** innate immune system, inflammatory bowel diseases, Crohn’s disease, FERAD ratio, p87, Adnab-9 antibody, Paneth cells, immunohistochemistry (IHC)

## Abstract

**Background:**

The perspective of inflammatory bowel disease (IBD) has changed radically since the first decade of the 21st century, and the formerly monolithic components of IBD, ulcerative colitis (UC), and Crohn’s disease (CD) have undergone a fundamental convergence, with realization that there is likely an element of shared pathogenesis. The ground shift began with genomic revelation but with the current emergence of the innate immune system (InImS) as a key player, allowing for improved understanding of the associations between the immune underpinnings of IBD.

**Methods:**

Using unique ferritin/fecal p87 (FERAD) or using colonoscopic effluent as denominator (FEREFF) and other ratios to test this hypothesis, we prospectively enrolled 2185 patients with increased risk of colorectal cancer, of whom 31 had UC and 18 CD, with 2136 controls and brought to bear in a convenient measure for the InImS, the FERAD ratio. The FERAD, FEREFF, and NLR ratios have been shown to be effective measures of the InImS in COVID-19 and various cancers. p87 is expressed in gut Paneth cells known to modulate the microbiome by secretion of alpha-defensins, a natural antibiotic. Other related parameters were also evaluated.

**Results:**

There was no significant difference between the FERAD ratio in UC and CD. However, differences between IBD entities and controls were substantial.

**Conclusions:**

InImS settings in IBD are similar between CD and UC. p87 tissue immunohistochemistry (IHC) is also shared. Other InImS markers, such as the absolute neutrophil/lymphocyte ratio, are also confluent between the two IBD forms.

## 1. Introduction

Ulcerative colitis (UC) was characterized in 1859 by Samuel Wilks, who called the disease idiopathic colitis, which was coined a year before Crohn’s disease (CD) in 1931 [1]. CD was reported in 1932 once Koch’s bacillus discovery enabled differentiation of Crohn’s from tuberculosis and the disease was first called regional ileitis [2]. Wilks’ postmortem case report on a 42-year-old woman who succumbed to IBD showed significant ileal inflammation, suggesting that the patient might have actually had CD. Thus, the controversy regarding an inter-relationship of CD and UC continues unabated since the earliest descriptions. There have been conversions and divergence in the treatment of IBD [3] with amino-salicylates felt to have better efficacy in UC but warranting further study in CD. Toward the close of the 20th century, it was felt that UC and CD were related disorders and likely shared susceptibility genes but have different immunoregulatory mechanisms [4]. In the following decade, the availability of single nucleotide polymorphisms and genome-wide association studies enabled detection of five shared susceptibility markers common to both diseases [5], leading to the notion of some shared etiology of UC and CD [6]. Since then, other disease commonalities have been shown to exist such as gastroduodenal lesions [7], an association of psoriasis [8], endodontic infection [9], commonality of treatment outcomes [10], and similar burden of disease [11]. Recently, much interest has been directed at the role of the InImS [12], and the relationship of FERAD to IBD is a major aim of our study, as will be described in the next section.

## 2. Results

**Figure 1.**
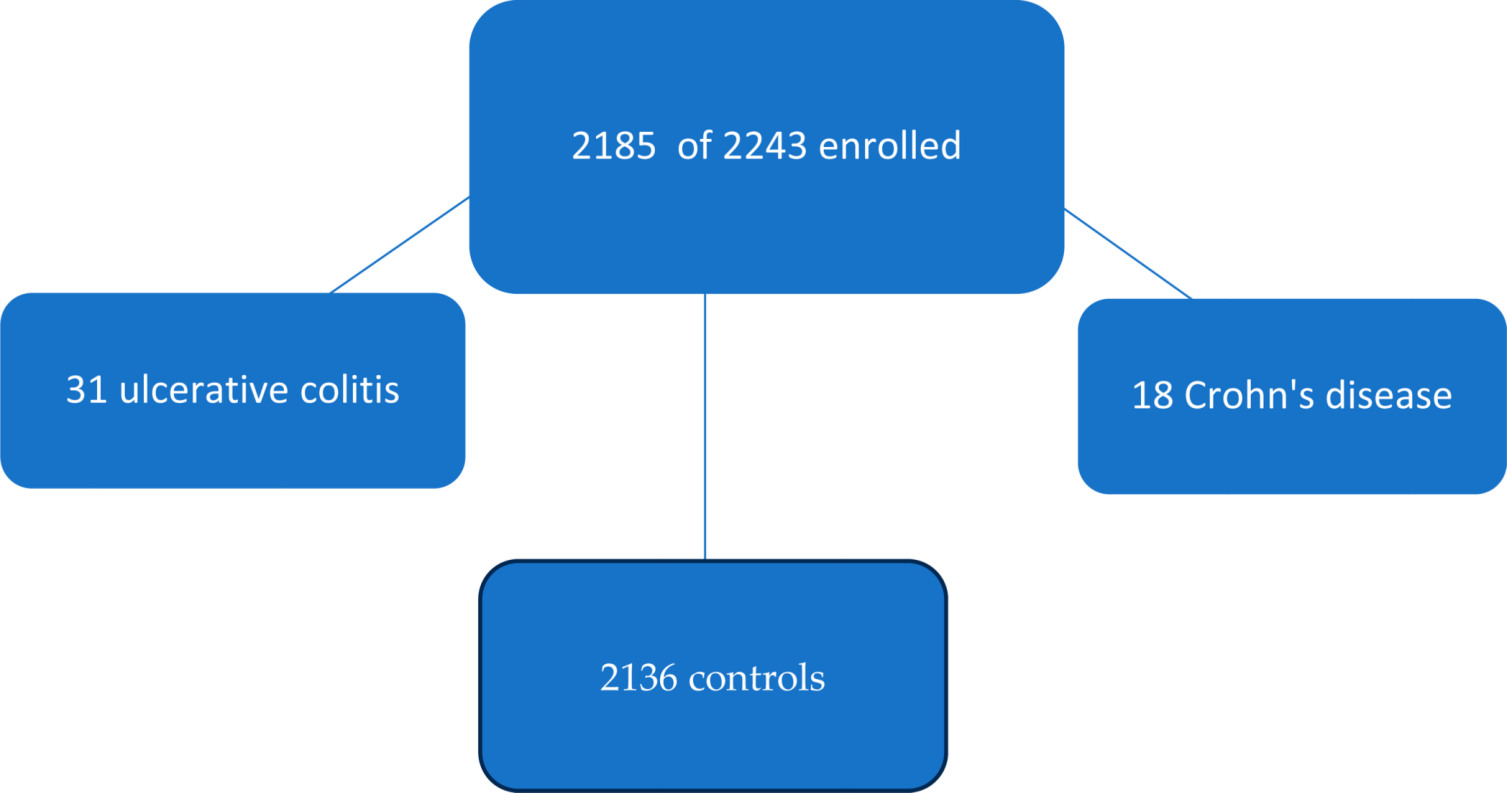
A flow diagram showing the total number and those of the IBD patients and controls.

A total of 2185 patients out of 2243 patients were selected for this study, of whom 31 had UC and 18 CD with 2136 controls with the flow diagram shown in Figure 1.

The demographic data of the participants are shown in Table 1.

The FERAD ratio, which is a measure of the InImS at the time of enrollment, is shown in Figure 1. The ratio appears not to be statistically significant when comparing UC to CD (*p* = 0.6), but both of these are extremely significantly different when comparing the FERAD ratio to controls.

**Figure 2.**
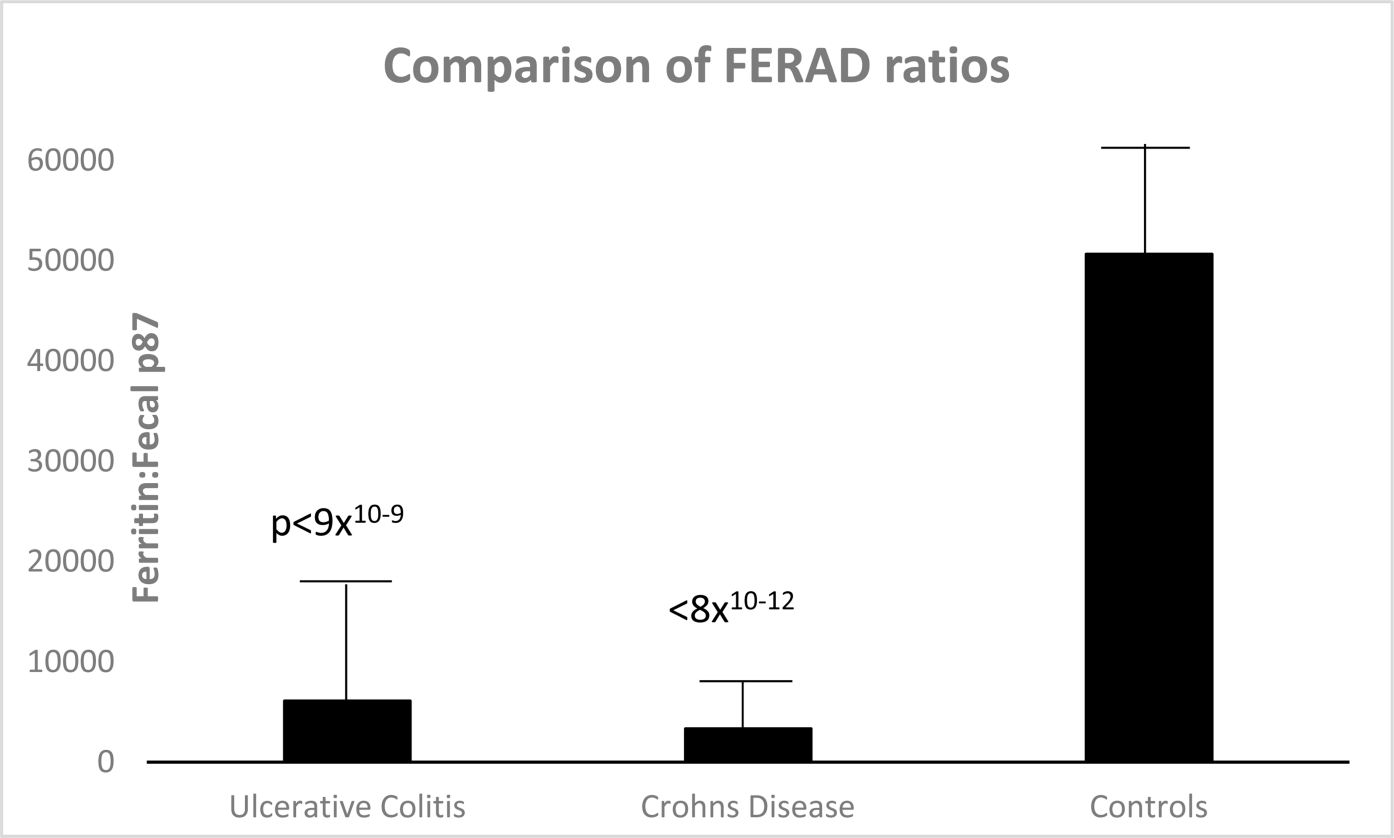
Bar diagram depicting the comparison of FERAD levels in IBD and controls.

As can be seen in Figure 2, the InImS as derived from the FERAD ratios is significantly lower in IBD compared to controls but are approximately equal in UC and CD, suggesting that the InImS is not operating at the expected levels, implying that the InImS is deficient in the response to these diseases.

We determined both fixed antigen by IHC and native antigen by ELISA with the results shown below in Table 2.

**Figure 3.**
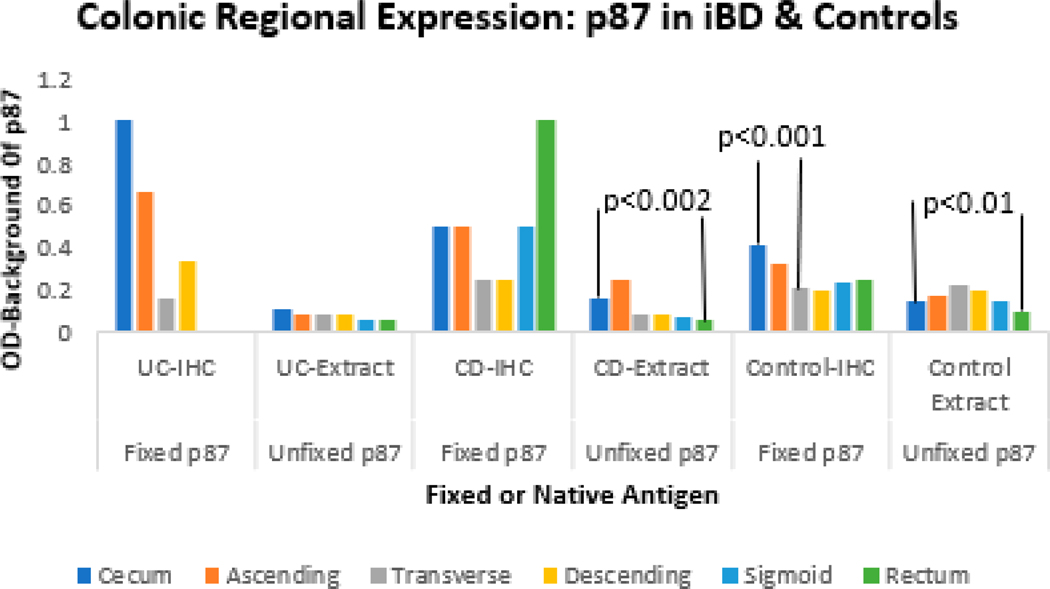
Bar diagram showing mean levels of colonic p87 expression in disease versus controls.

The above table summarizes the p87 expression of fixed tissues from six colonic regions by IHC and p87 unfixed extracted tissues from the same locations by ELISA. See Figure 3 below for depiction.

Typically, it is known that Paneth cells are predominantly found in the cecum and ascending colon. This is portrayed well by the UC IHC gradient from 1 and 0.66 in these two regions and tailing down to 0.2 and 0.3 in the transverse and descending colon with no p87 binding in the rectosigmoid regions in patients with UC where the rectosigmoid is invariably affected. There is a significant difference in the cecal extracts of CD patients versus rectal (*p* < 0.002). We have seen similar changes in the transverse colon regions when we investigated biotransformation in the colon, thought to be a neonatal effect persisting into adulthood [13]. An inverse relationship is seen in CD IHC where the rectum has the highest value tailing down to the lowest in the transverse colon, but this is not statistically significant. There is also no significant difference in UC as opposed to controls in the transverse colon regions. The p87 native antigen extracts, however, appear to be quite low in UC colon segments. In contrast, UC IHC and UC extracts are lowest in the left colon where disease predominates (NSS), but aside from a slightly depressed expression in the cecum, CD extracts follow a decreasing gradient from proximal to distal segments. Control IHC values are statistically significant when comparing cecum to the transverse colon (*p* < 0.001), and cecal extracts in controls are also significantly different from the rectal segment (*p* < 0.01). This could be the result of different treatments, such as steroid rectal suppositories, but these data are not available.

Other than the transverse colon trends’ lower expression in IHC UC and CD in the above figure, the other significant differences are in CD extracts and the controls. The actual statistics for those regions can be found in the summary in Table 2 in the first six entries. Overall, the p87 IHC expression patterns in UC IHC, CD IHC, and control IHC are in line with our previously reported data [14].

**Figure 4.**
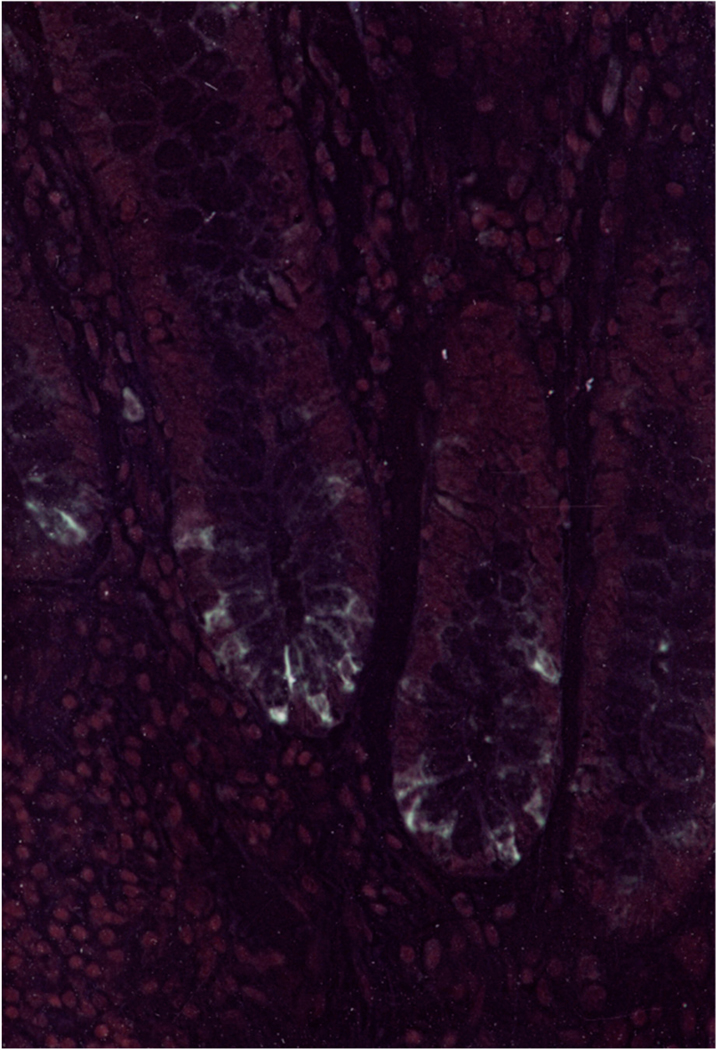
The crypts are well-oriented and likely represent inactive UC. The Adnab-9 antibody was used in a concentration of 1:50 dilution and developed using the ABC kit (see Section 3 below). The magnification in Figure 4 is 20×.

In Figure 4, an invert-phase photomicrograph depicts the presence of p87 using IHC in the colonic crypts of a patient with UC showing the typical distribution of Paneth cells in the colonic crypts.

Figure 2 shows a similar spread of p87 data in most of the colon with a different but similar rectosigmoid expression. The major finding is the significantly reduced p87 expression in the transverse colon.

By enumerating the total cumulation of adenomas, p87 Western blots, fecal p87, and extent of occult blood in the stool, we extended our observations to include additional neoplasia markers such as FEREFF, degree of neoplasia in the colon, and blood ferritin. We looked at smoking history as this can affect UC differently from CD. We also evaluated gastrointestinal symptoms, which were statistically significant in IBD versus controls and may suggest the degree of disease activity. Serum creatinine was important as it can reflect dehydration or, theoretically, effects of medication.

**Figure 5.**
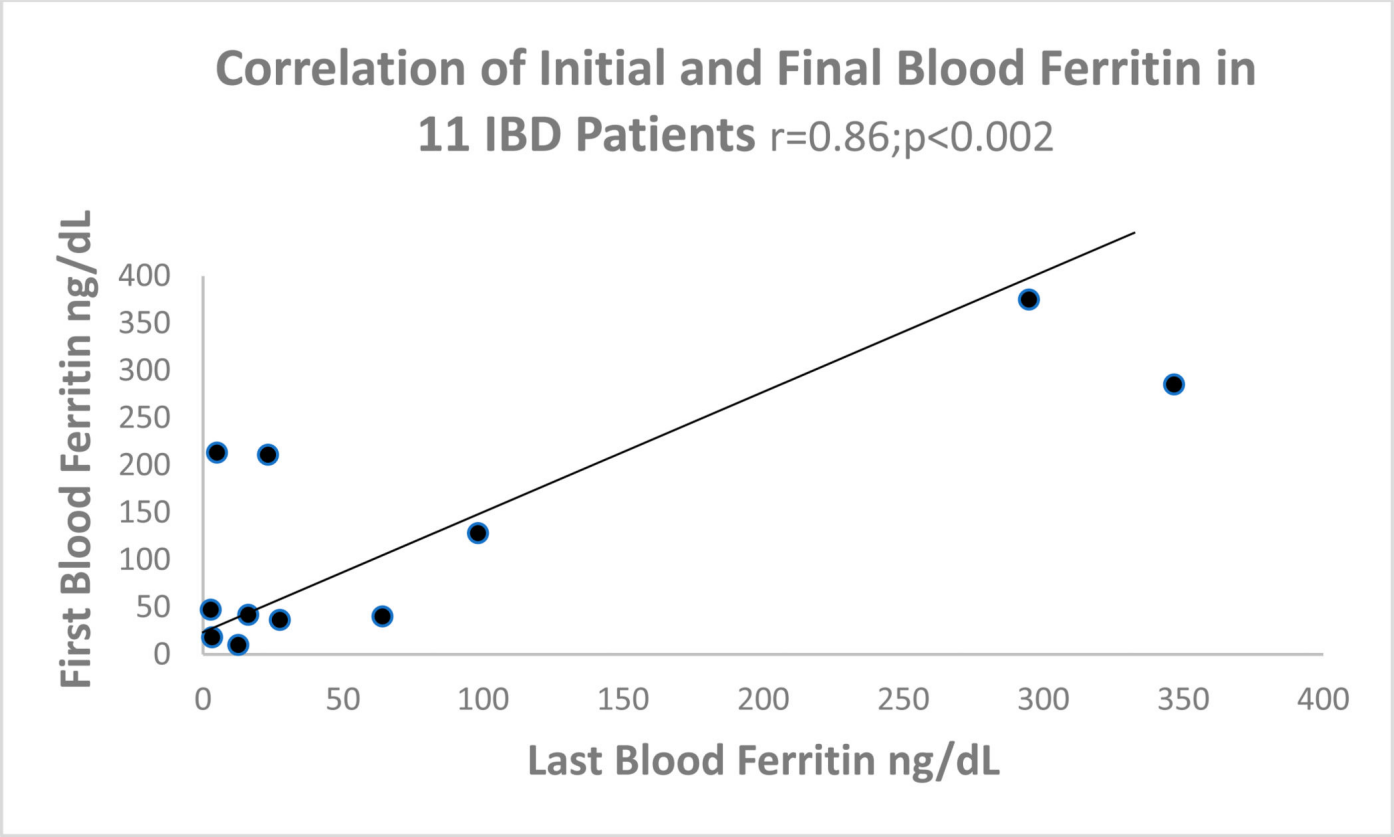
A direct correlation exists in IBD patients between initial and final ferritin determinations.

In Figure 5, in the correlation plot, it can be seen that there is a close association with ferritin levels when first taken and those last drawn. The mean time in years between the first and last blood drawings was 9.8 years.

## 3. Methods

The study design was a prospective, longitudinal observational research study. How the data were accumulated is as follows. The study coordinator set up an Excel spreadsheet and entered the demographics for each patient, which were static data and, aside from age, unlikely to change. The additional dynamic data were taken from the computerized record, which were primarily focused on colorectal neoplasia, but since IBD patients are at risk for neoplasia, ancillary data were entered such as family history, smoking, newly diagnosed neoplasia, etc. In time, an impressive array of data enabled us to obtain a comprehensive health picture of each participant. Most of the IBD patients were enrolled due to their increased cancer risk. Inclusion criteria included ability to provide consent and undergo colonoscopy, which was requested by the primary care physician. The computerized patient healthcare record was the source of the diagnosis of IBD upon chart review. Exclusion criteria were incomplete records or inability to provide informed consent or undergo colonoscopy. Diagnosis of IBD was based on clinical criteria, pathologic review, or specific biomarkers [14], but the latter was not available during the life of the study. Rather, concentrating on the InImS biomarker, we developed the FERAD ratio, derived by serum ferritin/fecal p87, which proved useful in the COVID-19 pandemic and the colorectal cancer marker study [14] and the commonly available [15] InImS absolute neutrophil/lymphocyte ratio (NLR), which bears a relationship to the FERAD ratio. Generally, disease grade and treatment were not recorded as the goal was to document the effect of screening using fecal parameters. The remainder of the patients enrolled, who had no IBD diagnosis, served as a control group. This lent some additional power to the study despite the fact that the IBD patient numbers were somewhat low but were in similar proportion to other limited number studies in the literature that measured novel biomarkers. The fecal p87 ELISA has been described [14] and will be briefly described. Consenting patients mailed 3 consecutive stool samples on 3 fecal occult blood test cards (Sure-Vue, Fisher HealthCare, Houston, TX, USA). Whole fecal protein used for standardization of plating in the ELISA was determined by the BCA kit (Thermo-Scientific Pierce, Waltham, MA, USA). A Vector ELISA kit (Vector Labs, Newark, CA, USA) was used with Adnab-9 (Dako Inc. Carpinteria, CA, USA) as the monoclonal recognizing the p87 antigen, which was plated as 5 μg protein/well in 96-well microtiter plates (Nunc, Copenhagen, Denmark), and for the negative controls, an irrelevant monoclonal with the same isotype as Adnab-9, UPC10 (Sigma, St. Louis, MO, USA) was used. Western blotting using 10% sodium Diacyl-Sulfate polyacrylamide gels, loaded using a standard protein concentration of 10 μg per lane in accordance with manufacturer instructions, and the gels were electrophoresed accordingly and transferred to polyvinylidene difluoride (PVDF) sheets (Kirkegaard and perry, Gaithersburg, MD, USA) and blocked with 5% milk solution. The aforementioned ABC kit was used to visualize specific bands, and Mr (relative mobility) was used to compute the molecular weights, using a protein ladder incorporated into the blot. Ferritin values were obtained from the computerized patient health record, where available, and were recorded with the dates obtained. The FERAD ratio was computed as follows: ferritin/fecal p87. The FEREFF ratio was computed as ferritin/p87 effluent (stool washings aspirated at colonoscopy) using the same ELISA methodology. In order to prove this hypothesis, components of this system would need to be examined at the tissue level using IHC for fixed antigen and extracted fresh tissue derived from tissue by colonoscopic biopsy and subjected to ELISA with the aforementioned protein standardization. The tissue was Dounce homogenized on ice, subjected to a low speed of 1000 rpm centrifugation, and the supernatant was fragmented by ultrasonic pulsation. The resultant sonoporated samples were subjected to a high-speed 10,000 rpm, and the supernatant was assayed for protein content and plated in a microtiter plate for an ELISA p87 estimation, as described above.

All patients provided written consent at time of enrollment in accordance with the VA clinical investigation committee and the Institutional Review Board of Wayne State University in Detroit, MI, USA.

The limitations of our study are that we do not have data regarding the therapy or severity of disease in our patients despite reporting symptomatology and blood present in the stool (Table 3), which are similar in both CD and UC, and the small number of patients with IBD enrolled in the study. Despite the relatively small numbers of IBD patients, we were able to garner sufficient data to gain a comprehensive picture of putative biomarkers. This was aided by strong numbers of controls.

## 4. Statistical Analysis

We used a statistical online package generously provided by Vassar Stats (https://vassarstats.net/ and last accessed on 27 December 2024. The values were tested for normality using the online Kolmogorov–Smirnov calculator https://www.socscistatistics.com/tests/kolmogorov/ accessed on 27 December 2024. Ordinal data were subjected to Student’s t-testing and non-ordinal data to χ^2^ analysis. Probability values at the <0.05 level were regarded as significant.

## 5. Discussion

For some time, it has been appreciated that the InImS in IBD is awry [16–18], centered in CD on nucleotide binding oligomerization domain 2 (nNOD-2), and innate immune responses are enhanced by Toll-like receptor 4 (TLR-4) ligand stimulation. Intestinal TLR9 is highly expressed in Paneth cells and, together with its ligand (CpG DNA), has a dual role in maintaining homeostasis and initiating inflammation. Normally NOD-2 enhances InImS response to CpG DNA, but this is lost in CD with homogenous NOD-2 mutations [18]. This pilot study gave credence to the use of lamina propria phagocytes in identifying specific microbiomes in IBD patients [19]. Other studiesinvestigating the use of miRNA and MRNA transcriptomes to delineate inflammatory pathways in IBD patients showed that the peripheral blood transcriptomes were broadly similar in UC and IBD [20] andin contrast, another microarray study showed miRNA differences using real-time PCR in saliva, blood, and colon in IBD patients and controls in 26 IBD patients compared to controls. A total of 10 CD and 6 UC patients showed mRNA differences [21], with just under half the number of patients in our current study.

The most plausible explanation for IBD pathophysiology is that of a luminal factor inciting an inadequate innate immune response, but the exact nature of the pathogenesis, which may be genetic, epigenetic, infectious, or immunologic, is not wholly understood, but miRNAs are favored [22]. Other researchers have examined chemical features of immune cytokine effectors in animal studies based on studies linked to abnormal factors in CRC with inappropriate activation of the InImS, centering on the role of neutrophils in helicobacter hepaticus-infected Rag2(−/−) mice [23]. In that study, comparison to the human shows that neutrophil signatures in UC sera favor UC, and both neutrophil and macrophage activity was found in CD. Specific polymorphisms in the interleukin system such as IL-23 are found both in UC and CD, and, to complicate matters further, IL-23 receptors have been found in innate lymphoid cells that have both pro- and protective inflammatory activity to commensal or pathogenic bacteria, prompting caution when considering novel therapeutic intervention [24,25]. These two last cited papers suggest that the ongoing inflammation of UC and CD share common effectors, and this supports our hypothesis that there is a plurality of evidence suggesting shared mechanisms of inflammation. Interestingly, immunoglobulin isotypes such as Ig1 are more active in UC, producing a great respiratory burst in response to specific bacterial surface antigens, whereas in CD, the response is of the IgG2 isotype [26]. Another conundrum is the failure of certain patients with IBD, both UC and CD, to respond to treatment directed toward anti-tumor necrosis factor (TNF), and new biomarkers are needed to differentiate such patients [27]. Defects in the immune system seem to be at the forefront of IBD causation, and it has been established that the innate mucosal barrier in ileal CD, in which alpha-defensins from ileal Paneth cells are secreted, are impacted by Paneth cell defects, and in colonic CD, beta-defensins in CD are lacking. In UC, the mucus protective layer, which protects against the bacterial load, is also lacking due to reduced differentiation of stem cells to goblet cells. Our paper introduces the FERAD and FEREFF ratios, which likely incorporate measures of the InImS and suggest that these ratios are significantly reduced as compared to controls in Figure 2 and lower levels of p87 immunohistochemistry in the transverse colon of UC compared to CD (Table 2 and Figure 3). These differences are greatly magnified when compared to controls and likely reflect the innate immune deficiencies covered in this section. We have shown that the ferritin enumerator of both FERAD and FEREFF ratios is fairly constant over time and unlikely to be much altered (Figure 5). However, we reported that FERAD ratios can be manipulated by altering p87 using turmeric to increase p87 and folic acid to decrease this denominator [28].

In addition, a recent study linking fatty liver diseases to IBD [29] reports that the increase in incidence affects both UC and CD, suggesting a final common pathway for both entities in diverse organs affected by inflammation. This further supports our contention of a partially shared disease pathogenesis, and the fact that similar agents are used for treatment further consolidates our viewpoint. The FERAD ratio as a gauge of historic InImS activity, similar in both UC and CD, can be manipulated by common low-risk supplements, such as turmeric and folates, and can be incorporated into the treatment armamentarium at low cost and with few safety concerns.

Recently, the actions of the fecal virome with the bacterial microbiome in IBD has been studied, which also incorporates the InImS [30], and features a biological tug-of-war between anti-inflammatory resident colon viruses and pro-inflammatory IBD viromes, which may allow for novel therapy and intervention. In addition, melanocortins are post-translational peptides that salubriously activate innate immune effectors, are mediated by NF-κB modulation, and are found in inflamed tissue but not in normal mucosa using IHC in humans with IBD [31], exciting expectations for additional medical intervention.

Our study investigated the nuances of the InImS in IBD and its novel biomarkers, FERAD, FEREFF, and p87, and provided some justification for the inclusion of ferritin in the FERAD ratio, which would not have been apparent due to the “common wisdom”. While the intertwining of IBD and cancer is an unfortunate reality, we believe that with additional studies we may be able to tease these apart and provide ideal biomarkers to guide treatment and early detection of neoplasia.

## Figures and Tables

**Table 1.** Demographics of the enrollees.

Parameter	Ulcerative Colitis n = 31	Crohn’s Disease n= 18	Controls n = 2136	*p*-Value

Age (years)	62.1 ± 14.5 *	51.4 ± 13.6 ˆ	60.0 ± 12.3 *	* NSS; ˆ < 0.013
Sex M/F %	M26/F5 83.9%	M15/F3 83.3%	M1904/F232 89.1%	NSS
Ethnicity aa	13aa/18c 41.9%	8aa/10c 44.4%	1185aa/951c 55.5%	NSS

Age given in mean ± standard deviation; sex M—male; F—female; NSS—not statistically significant; aa—African American; c—Caucasian;

ˆ —ages of CD patients were significantly different when compared to controls; and

* —NSS.

**Table 2.** A comparison of p87 immunohistochemistry in fixed sections and p87 ELISA in tissue extracts.

Parameter	UC n = 31	CD n = 18	Cnt n = 2136	p UC vs. CD	p UC vs. Cnt	p CD vs. Cnt
p87ihc Cec	1 ± 1	0.5 ± 0.707	0.411 ± 0.669	0.79	0.134	0.85
p87ihc Asc	0.667 ± 0.577	0.5 ± 0.707	0.329 ± 0.618	0.79	0.35	0.69
p87ihc trans	0.167 ± 0.289	0.250 ± 0.354	0.209 ± 0.471	0.79	0.9	0.9
P87ihc Desc	0.333 ± 0.577	0.500 ± 0.707	0.201 ± 0.478	0.87	0.64	0.89
p87ihc Sigm	0	0.500 ± 0.707	0.235 ± 0.532	0.27	0.45	0.5
p87ihc Rectu	0	1.000 ± 1.414	0.247 ± 0.546	1	0.44	0.06
p87Elisa Cec	0.110 ± 0.092	0.165 ± 0.008	0.153 ± 0.157	0.48	0.64	0.92
p87Elisa Asc	0.089 ± 0.351	0.255 ± 0.030	0.179 ± 0.244	0.9	0.79	0.66
p87Elisa Tran	0.087 ± 0.291	0.088 ± 0.005	0.230 ± 0.420	0.61	0.95	0.63
p87Elisa Desc	0.082 ± 0.101	0.083 ± 0.074	0.194 ± 0.267	0.9	0.52	0.56
p87Elisa Sig	0.065 ± 0.570	0.077 ± 0.022	0.154 ± 0.212	0.58	0.155	0.61
p87Elisa Rect	0.055 ± 0.005	0.066 ± 0.022	0.101 ± 0.113	0.06	0.23	0.66

Cnt—control; Cec—cecum; Asc—ascending colon; trans—transverse colon; Desc—descending; Sigm—sigmoid colon; and Rect—rectum.

**Table 3.** We contrasted a number of important parameters to determine colorectal neoplasia.

Parameter	UC n = 31	CD n = 18	Cnt n = 2136	p UC vs. CD	p UC vs. Cnt	p CD vs. Cnt
Total Ads	0.968 ± 1.602	0.316 ± 0.749	2.060 ± 3.754	0.058	<0.001	<4 × 10−9
Western Blot+	4 + 16—20%	9 + 9—50%	98 + 628—13.5%	=0.087	0.6 NSS	6.41 (2.48–16.54) < 0.0004
P87 stool+	11 + 16—39.3%	2 + 9—18.2%	212 + 678—23.8%	0.3 NSS	2.2 (1.01–4.81); *p* < 0.044	0.8 NSS
FOBT+	11 + 19—36.7%	4 + 12—25%	200 + 1001— 16.7%	0.3 NSS	2.9 (1.36–6.18) < 0.009	0.9 NSS
Effluent p87	0.215 ± 0.235	0.042 ± 0.061	0.202 ± 0.356	0.08	0.9	<3 × 10^−6^
FEREFF	0.022 ± 0.029	0.027 ± 0.065	0.003 ± 0.006	0.9	<0.00000071	<0.0000006
Degree Neo	0.310 ± 0.604	0.235 ± 0.664	0.723 ± 0.886	0.7	<0.013	<0.024
Ferritin Se	143.9 ± 167.6	71.84 ± 93.08	192.4 ± 248.3	0.2	0.6	<0.0009
Smokers	3 + 23—11.5%	5 + 9—35.7%	468 + 742—38.7%	0.1 NSS	0.21 (0.06–0.69) <0.009	1 NSS
Symptoms%	27 + 4—87.1%	18 + 1—94.7%	1035:867 54.4%	0.6 NSS	5.65 (1.97–16.22) < 0.0006	15.08 (2.01–113.18) < 0.002
Serum Cr	1.111 ± 0.240	1.010 ± 0.192	1.29 ± 1.950	0.08	<0.02	<0.0006

Cnt—Controls; Ads-adenomas; NSS—not statistically significant; FEREFF is ferritin: colonic effluent ratio; Neo—neoplasia; Se—serum; and Cr—creatinine. Other NSS parameters were obesity, vitamin D levels; type-2 diabetes mellitus; mortality; family history of cancer; alcohol intake; illicit drugs; hepatitis C; proton pump inhibitors; non-steroidal anti-inflammatory medications; and individuals with cancer. Where applicable, χ^2^ analysis outcomes were presented with odds ratio and confidence intervals, and where applicable, *p*-values were included. Symptoms were confined to those likely emanating from the gastrointestinal tract.

## Data Availability

The data presented in this study are available upon request from the corresponding author under the Data Transfer Agreement and all the following conditions apply. An application must contain statements to the effect of the following: What data are being requested; what data may be used; who will access the data; and how the data will be accessed; stored and safeguarded. The applicant must also address how the data will be disposed of, after completion of the data review. Suggested data availability statements are available in the VHA directive 1200.12 0f 3/9/2209. The VHA Handbook addresses both the use of the data for research and the clinical and administrative data repositories for research. It also addresses the development and use of the data research repositories.
